# Matrix-Assisted Laser Desorption/Ionization Mass Spectrometry to Detect Diagnostic Glycopeptide Markers of Congenital Disorders of Glycosylation

**DOI:** 10.5702/massspectrometry.A0084

**Published:** 2020-04-23

**Authors:** Yoshinao Wada

**Affiliations:** 1 Osaka Women's and Children's Hospital (OWCH), 840 Murodo-cho, Izumi, Osaka 594–1101, Japan

**Keywords:** congenital disorder of glycosylation, diagnosis, screening, glycopeptide

## Abstract

Congenital disorders of glycosylation (CDG), an increasingly recognized group of diseases that affect glycosylation, comprise the largest known subgroup of approximately 100 responsible genes related to *N*-glycosylation. This subgroup presents various molecular abnormalities, of either the CDG-I or the CDG-II type, attributable to a lack of glycans or abnormal glycoform profiles, respectively. The most effective approach to identifying these *N*-glycosylation disorders is mass spectrometry (MS) using either released glycans, intact glycoproteins or proteolytic peptides as analytes. Among these, MS of tryptic peptides derived from transferrin can be used to reliably identify signature peptides that are characteristic of CDG-I and II. In the present study, matrix-assisted laser desorption/ionization (MALDI) MS was applied to various *N*-glycosylation disorders including ALG1-CDG, B4GALT1-CDG, SLC35A2-CDG, ATP6V0A2-CDG, TRAPPC11-CDG and MAN1B1-CDG. This method does not require the prior enrichment of glycopeptides or chromatographic separation, and thus serves as a practical alternative to liquid chromatography-electrospray ionization MS. The signature peptides are biomarkers of CDG.

## INTRODUCTION

**Figure 1:**
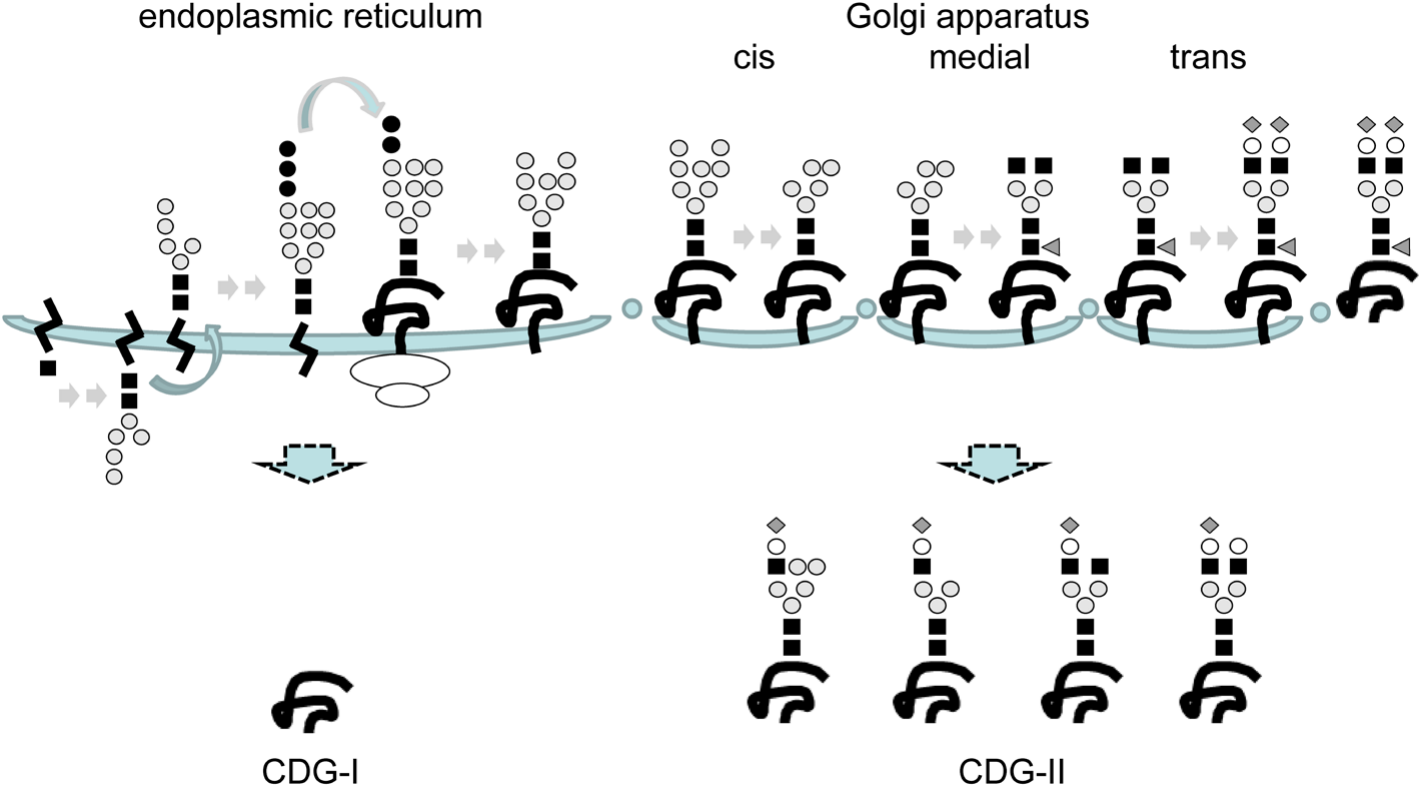
Fig. 1. Simplified depiction of the pathway for *N*-glycosylation biosynthesis. Abnormal molecules and glycoforms produced in CDG are illustrated below the pathway.

Congenital disorders of glycosylation (CDG) are an expanding group of disorders caused by defects in the glycosylation of proteins, and include more than 125 known genetic diseases.^1,2)^ Among the various forms of CDG, those affecting *N*-glycosylation constitute a major subgroup, which is characterized by two types of molecular defects, *i.e.*, a lack of glycans (CDG-I) or an abnormal glycan profile (CDG-II)^3)^ (Fig. 1). CDG-I is caused by a defect in the synthesis of a lipid-linked glycan and the subsequent *en bloc* transfer of glycans to nascent proteins in the endoplasmic reticulum (ER). The defects of CDG-II occur in the subsequent trimming and decoration steps to form mature glycoforms in the ER and Golgi apparatus. In both types, patients also have unaffected glycoproteins, because the complete loss of mature glycans is incompatible with life.

In 1992, mass spectrometry (MS) revealed a molecular abnormality derived from a defect in the early step of the *N*-glycosylation of transferrin, and this observation contributed to establishing CDG as an entity.^4)^ In the last thirty years, nearly 100 different disease types or genes responsible for congenital disorders of *N*-glycosylation have been identified.^5)^ Since the symptoms and signs of CDG are quite diverse, making it impossible to establish a diagnosis using routine laboratory tests,^6,7)^ MS is an essential approach for accurately diagnosing CDG.^8)^

MS can use either intact transferrin, glycans or glycopeptides as the analyte for detecting CDG,^1,9,10)^ and each approach has its own advantages and disadvantages. An analysis of released glycans is well-established and applicable not only to transferrin but also to whole serum proteins. The method requires multiple sample preparation steps including the release of glycans from glycoproteins and subsequent derivatization procedures such as permethylation. MS is performed with either electrospray ionization (ESI) or matrix-assisted laser desorption/ionization (MALDI).^11)^ MS of glycans is unable to detect CDG-I, since there are no alterations in the glycoform. MS of intact transferrin (*M*_r_ 79,556),^10)^ which is modified by the attachment of two complex-type sialylated glycans with minimal microheterogeneity, can be rapidly analyzed, since only immunopurification is needed before loading onto on-line liquid chromatography (LC) MS. ESI of intact transferrin generates ions with 25–45 charges, which requires a wide mass range up to *m*/*z* 3000. In addition, parameters must be carefully set for both the measurement and spectral deconvolution in order to identify low-intensity diagnostic signals that might be buried in the chemical noise or lost below the cut-off threshold. Such problems and pitfalls associated with the analysis of intact transferrin have been discussed in detail in a previous report.^12)^ On the other hand, MALDI-MS of intact transferrin is effective for identifying CDG-I,^13)^ but the resolving power is not sufficient to detect the altered glycoform profiles that are characteristic of CDG-II.

MS of glycopeptides is a standard proteomic method. The tryptic digestion of transferrin followed by MS has been used to characterize the immature glycoforms of CDG-II,^14,15)^ but this approach is not widely employed for CDG screening. Barroso *et al.* recently reported a capillary LC-ESI-MS method to identify CDG and to discriminate between various CDG-II types based on the relative abundance of signature ions.^16)^ We report herein that the MALDI-MS of tryptic peptides derived from transferrin is a viable alternative to LC-ESI-MS, and the peptide masses can be useful for diagnosing CDG-I and -II serving as reliable biomarkers of CDG.

## MATERIALS AND METHODS

### Patients

Anonymized serum samples were delivered to OWCH to screen patients for suspected CDG. The genetic diagnosis was made before or after identifying the molecular abnormality by MS.

### Sample preparation and MALDI-MS

MS of glycopeptides for glycoform profiling was performed according to a previously reported method, with minor modifications.^12)^ Briefly, an affinity column was prepared using a rabbit polyclonal antibody against human transferrin (DAKO, Denmark) and a ligand-coupling Sepharose column (HiTrap NHS-activated HP, GE Healthcare, Piscataway, NJ, USA), and the antibody-coupled Sepharose was recovered from the column. Ten μL of serum or plasma were mixed with a 20-μL slurry of Sepharose in 0.5 mL of phosphate-buffered saline and the resulting solution incubated at 4°C for 30 min. After washing the Sepharose, the transferrin was eluted in 0.1 M glycine–HCl buffer at pH 2.5. The purified transferrin was dissolved in 0.5 mL of 6 M guanidium hydrochloride, 0.25 M Tris–HCl, pH 8.5 and reduced by treatment with 5 mg of dithiothreitol at 60°C for 30 min. A 10 mg portion of iodoacetamide was then added to achieve carbamidomethylation, and the resulting solution was incubated in the dark at room temperature for 30 min. The reagents were removed by a NAP-5 gel filtration column (GE Healthcare) equilibrated with 0.05 N HCl, and the recovered protein solution was adjusted to pH 8.5 with Tris. Digestion was performed using a mixture of trypsin (Sequencing Grade Modified Trypsin, Promega, Madison, MI, USA) and *Acromobacter* lysylendopeptidase (Wako, Japan) at 37°C for 12 h. Neither enrichment nor the purification of glycopeptides was carried out. The digest was desalted using a Millipore ZipTip C18 pipette tip and analyzed with a MALDI time-of-flight (TOF) mass spectrometer equipped with a 337-nm wavelength nitrogen laser (Voyager DE-Pro, SCIEX, Framingham, MAA). The sample matrix was 20 mg/mL of 2,5-dihydroxybenzoic acid dissolved in 50% acetonitrile in water. Measurements were performed for positive ions, and both linear and reflectron TOF modes were used.

## RESULTS AND DISCUSSION

**Figure 2:**
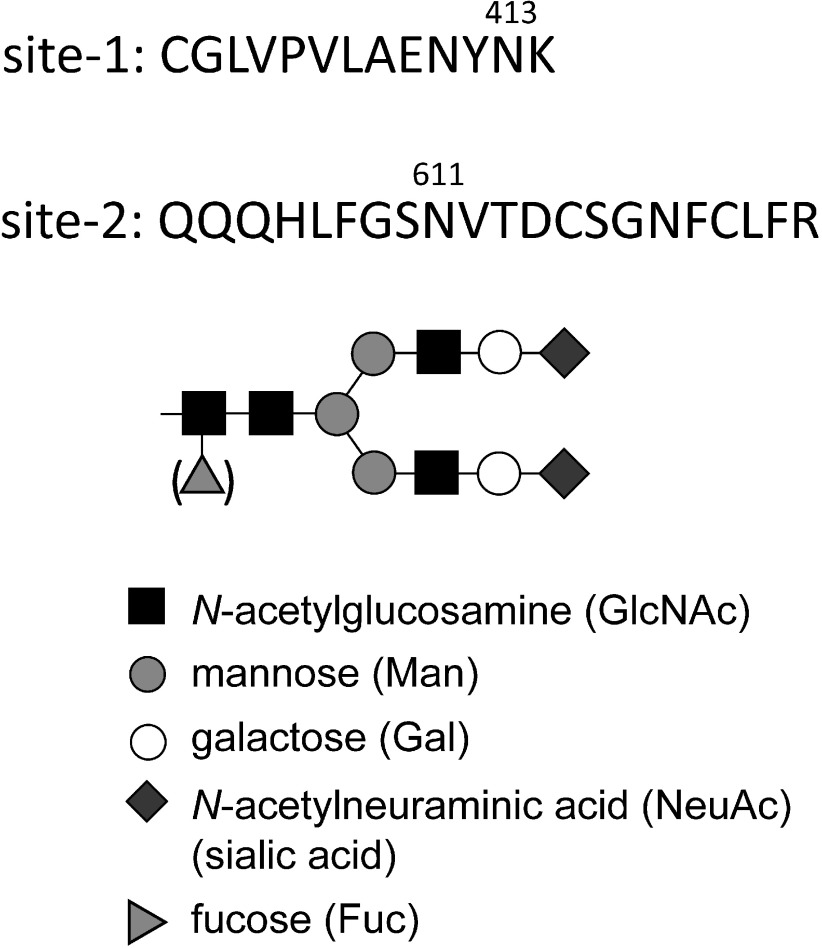
Fig. 2. Sequences of the tryptic glycopeptide and the major glycoforms of transferrin.

**Figure 3:**
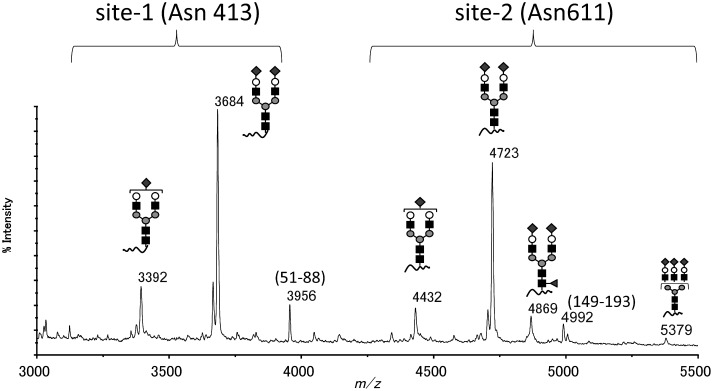
Fig. 3. MALDI linear TOF mass spectrum of tryptic peptides of transferrin obtained from a healthy individual. The peaks indicated by sequence numbers in parentheses are ions without glycosylation sites. Their sequences are AIAANEADAVTLDAGLVYDAYLAPNNLKPVVAEFYGSK (51–88) and AVANFFSGSCAPCADGTDFPQLCQLCPGCGCSTLNQYFGYSGAFK (149–193). Glycoforms at site-1 and site-2 are displayed in the lower and higher mass regions, respectively.

Transferrin is abundant (approximately 2 mg/mL) in serum. It contains two *N*-glycosylation sites at Asn413 and Asn611 (Fig. 2), which are alternatively named Asn432 and Asn630, respectively, of the preproprotein. The glycoform is comprised mainly of a sialylated biantennary form with little microheterogeneity, making transferrin suitable for screening for glycosylation disorders. The molecular weights of these tryptic peptides of 13 and 21 amino acid residues exceed 3,000 when a sialylated biantennary glycan of 2,206 Da is attached. There are only a few other peptides larger than 3,000 Da in the tryptic digest of transferrin (see the legend of Fig. 3), thus allowing the MALDI-based approach to skip glycopeptide enrichment.

### Characteristics of MALDI MS of glycopeptides

A MALDI linear TOF mass spectrum of transferrin from a healthy individual is shown in Fig. 3. Attachment of glycans is generally thought to impair ionization efficiency and thus, the signal intensity of the peptides. Interestingly, however, the intensities of glycopeptides in this mass spectrum are not smaller than those of other unglycosylated peptides. This is probably due to the acidity of the latter peptides (see the sequences shown in the figure legend). In the mass spectrum, the relative intensities of glycopeptide ions bearing one or two sialic acids (*N*-acetylneuraminic acids) do not actually reflect the true molar ratio, and the peak for the mono-sialylated species is enhanced as compared with that of the di-sialylated form.^11)^ This phenomenon is often more evident for the glycopeptide of site-1 than that of site-2. A small signal at *m*/*z* 4868.9 corresponds to a fucosylated glycoform at site-2, which is barely detectable at site-1.

### CDG-I

**Figure 4:**
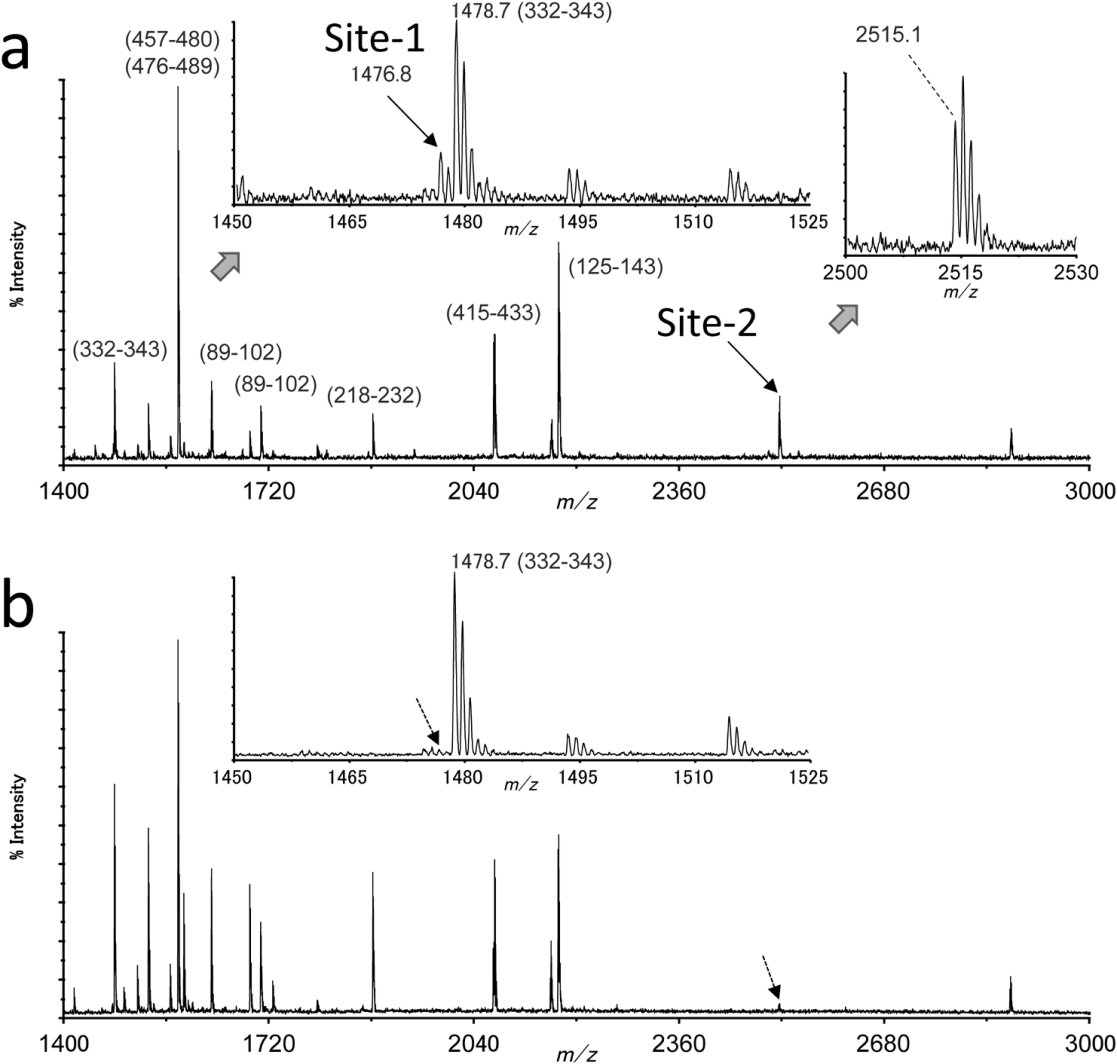
Fig. 4. MALDI reflectron TOF mass spectra of tryptic peptides of transferrin. (a) CDG-I patient. Arrows indicate diagnostic ions. This patient is a compound heterozygote for *ALG1* mutations and has a mutation in the *SSR4* gene which is also among the candidate causes of CDG-I type abnormalities. (b) Healthy individual. Broken arrows indicate the positions of diagnostic ions; it is noteworthy that a small peak at *m*/*z* 2525.1 is observed in this unaffected subject.

The molecular phenotype of CDG-I is characterized by the incomplete occupancy at the *N*-glycosylation sites. MALDI reflectron TOF mass spectra from a CDG-I patient (ALG1-CDG^17)^) and a healthy individual are shown in Figs. 4a and 4b, respectively. In the patient, the unglycosylated ions at *m*/*z* 1476.8 and *m*/*z* 2515.1 indicate the absence of glycosylation at site-1 and site-2, respectively (Fig. 4a). A small signal corresponding to the missing glycosylation at site-2 is observed in some healthy individuals (Fig. 4b). This is not derived from an artifact during sample preparation or the MALDI process and is reproducible for the same serum sample. The glycoform profile is unaffected in CDG-I (data not shown).

### CDG-II

CDG-II is caused by a defect in the processing or maturation of glycans. The trimming of terminal sugars occurs in the ER and monosaccharides such as *N*-acetylglucosamine, galactose, *N*-acetylneuraminic acid and fucose are then sequentially added to produce mature glycoforms in the Golgi apparatus. A defect in this process generates immature or abnormal glycoforms (Fig. 1). The genes responsible for CDG-II are not only the enzymes directly catalyzing the removal or addition of sugars but also the molecules that are involved in vesicular transport or organellar homeostasis, which expands the CDG category.

**Figure 5:**
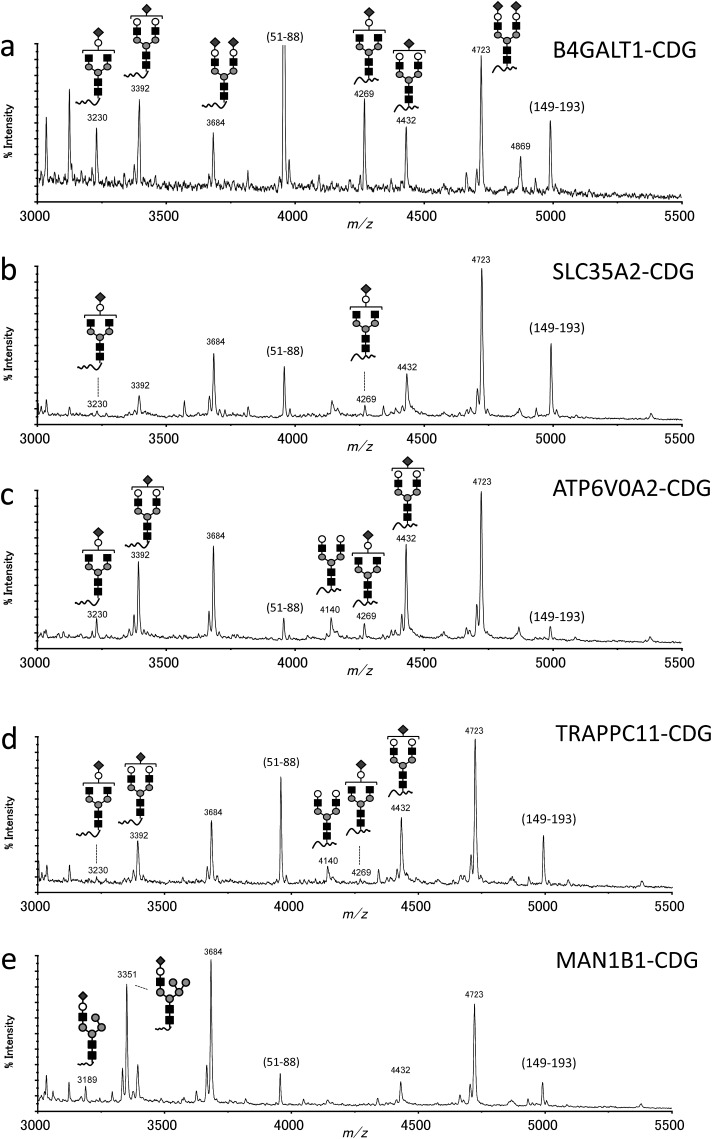
Fig. 5. MALDI linear TOF mass spectrum of tryptic peptides of transferrin from patients with various types of CDG-II.

The MALDI mass spectra of various types of CDG-II are presented in Fig. 5. B4GALT1-CDG is a typical CDG-II involving a Golgi enzyme deficiency.^18)^ An impaired activity of β-1,4-galactosyl transferase results in the appearance of the glycoform lacking galactose (Fig. 5a). For unknown reasons, the monosialylated, but not the galactosylated ions at *m*/*z* 3392 and *m*/*z* 4432, were also increased in this patient.

(a) B4GALT1-CDG. (b) SLC35A2-CDG. (c) ATP6V0A2-CDG. (d) TRAPPC11-CDG. (e) MAN1B1-CDG.

SLC35A2 is the Golgi-localized UDP-galactose transporter. A mutation in *SLC35A2* results in the generation of galactose-deficient glycoforms (Fig. 5b), similar to B4GALT1-CDG. The diagnostic mono-galactosylated ions at *m*/*z* 3230 and *m*/*z* 4269 were present at low levels but were clearly detected, while they were not observed in healthy individuals (Fig. 3). In reported cases with severe molecular phenotypes, glycopeptide ions without galactose, which should be observed at *m*/*z* 3068 and *m*/*z* 4107, were present as well.^19,20)^ In contrast to B4GALT1-CDG, the glycoforms lacking a single sialic acid at *m*/*z* 3392 and *m*/*z* 4432 were not increased.

ATP6V0A2-CDG is caused by mutations in the gene encoding the a2 subunit of the vacuolar H^+^-ATPase (V-ATPase).^21)^ Although the precise mechanism underlying the effects of mutations of this gene on glycosylation remains unclear, alterations of the localization of glycosyltransferases in the Golgi apparatus and changes in their enzymatic activity have been suggested to be involved.^22)^ As shown in Fig. 5c, decreased sialylation (*m*/*z* 3392, *m*/*z* 4140 and *m*/*z* 4432) was more evident than decreased galactosylation (*m*/*z* 3230 and *m*/*z* 4269).

Mutations in *TRAPPC11*, a subunit of the TRAPPIII complex, resulted in a delay of vesicular transport in the Golgi apparatus.^23)^ The MALDI mass spectrum of TRAPPC11-CDG showed a significant decrease in the extent of sialylation (*m*/*z* 3392, *m*/*z* 4140 and *m*/*z* 4432) as well as low-intensity signals indicating decreased galactosylation (*m*/*z* 3230 and *m*/*z* 4269) (Fig. 5d), which was consistent with observations of an earlier report.^23)^

Regarding sialylation, the level of sialylation to be judged “abnormal” are statistically defined, because the mono-sialo and even asialo species are present in healthy individuals and are enhanced in the MALDI mass spectrum.^11)^ In this study, the ratio of the intensities for site-2 glycopeptide ions at *m*/*z* 4432 and *m*/*z* 4723 was 0.16±0.02 (mean±S.D., *n*=5) in unaffected individuals. The ratio was significantly elevated for B4GALT1-CDG (0.45), ATP6V0A2-CDG (0.67) and TRAPPC11-CDG (0.46), the only exception being SLC35A2-CDG (0.16), in these mass spectra. It should be noted that the ratio depends on the instrumental parameters involved in the analysis.

As shown by the cases described above, most CDG-II types have a decrease in sialylation and/or galactosylation in common as a molecular feature, although a lack of *N*-acetylglucosamine caused by a deficiency in GlcNAc transferase II (MGATII-CDG) has also been reported.^24)^ An exception is MAN1B1-CDG caused by an α-mannosidase I deficiency,^25)^ which removes α-1,2 mannose units in the Golgi apparatus and is necessary for the formation of complex-type glycans. The hybrid-type glycoforms observed at *m*/*z* 3189 and *m*/*z* 3351 might be specific biomarkers for MAN1B1-CDG (Fig. 5e). Interestingly, these glycoforms were not observed at site-2 in the mass spectrum, probably due to the poor accessibility of this enzyme to the site-2 glycan.

Finally, it should be kept in mind that these molecular abnormalities may diminish depending on non-genetic factors such as age, nutrition and treatment. Furthermore, the qualitative or quantitative approach described herein does not allow identification of some types of CDG such as MOGS-CDG^26)^ and SLC35C1-CDG,^27)^ which have an apparently normal transferrin profile. Fucosylation levels in the latter case are effectively evaluated by the MS of immunoglobulin-G,^11)^ and the MS-based method to detect MOGS-CDG will be described elsewhere. Nevertheless, determining the signature ions of tryptic peptides of transferrin, as shown in Table 1, is useful as an initial laboratory approach for patients who are suspected to have CDG. These diagnostic markers will allow technicians, even those without expertise in glycobiology, to effectively screen patients for CDG in the clinical laboratories.

**Table 1:** Table 1. Diagnostic peptides and *m*/*z* values.

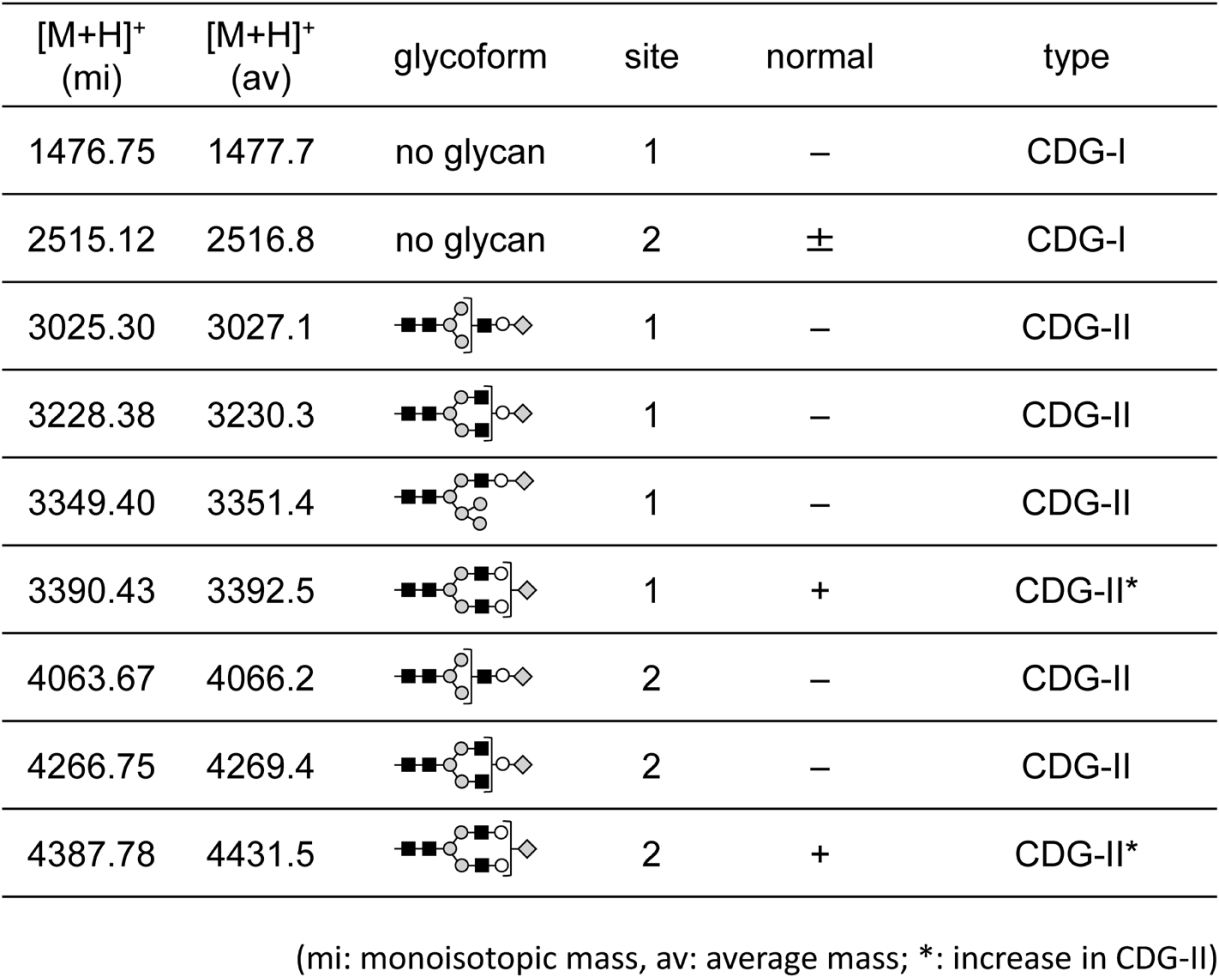

## CONCLUSION

MALDI-MS of tryptic digests of transferrin offers an easy and reliable method for first-line screening for CDG. Although the *N*-glycan structure is complicated, the number of key ions are limited, thus facilitating the detection of CDG. Each laboratory should determine its own normal range for these biomarkers especially for the sialylation levels.

## Notes

Mass Spectrom (Tokyo) 2020; 9(1): A0084
